# The role and possible mechanism of the long noncoding RNA LINC01260 in nonalcoholic fatty liver disease

**DOI:** 10.1186/s12986-021-00634-4

**Published:** 2022-01-12

**Authors:** Xiaoxiao Ge, Tao Sun, Yanmei Zhang, Yongqing Li, Peng Gao, Dantong Zhang, Bingyang Zhang, Peijun Wang, Wanshan Ma, Sumei Lu

**Affiliations:** 1 Department of Laboratory Medicine, Shandong Provincial Qianfoshan Hospital, Shandong University, Jinan, 250014 Shandong People’s Republic of China; 2 Department of Laboratory Medicine, The First Affiliated Hospital of Shandong First Medical University, Jinan, 250014 Shandong People’s Republic of China; 3 Medical Research Center, The First Affiliated Hospital of Shandong First Medical University, Jinan, 250014 Shandong People’s Republic of China; 4 Blood Transfusion Department, Henan Provincial People’s Hospital, People’s Hospital of Zhengzhou University, Zhengzhou, 450003 Henan People’s Republic of China; 5 Department of Clinical Laboratory, The Affiliated Hospital of Shandong University of Traditional Chinese Medicine, Jinan, 250014 Shandong People’s Republic of China

**Keywords:** Nonalcoholic fatty liver disease, Long-chain noncoding RNAs, Eukaryotic circular sequencing, Oleic acid, Hepatocyte steatosis

## Abstract

**Objective:**

To investigate the differential expression profile of lncRNAs in the nonalcoholic fatty liver disease (NAFLD) model induced by oleic acid (OA) and to further explore the role of LINC01260 (ENST00000255183) in NAFLD, providing theoretical support for the clinical value of lncRNAs in NAFLD.

**Methods:**

OA (50 μg/mL) was used to induce steatosis in normal human LO2 hepatocytes for 48 h and was verified by Oil red O staining. Differential expression profiles of lncRNAs were obtained by eukaryotic circular sequencing (RNA/lncRNA/circRNA-seq) techniques. A gain-of-function (GOF) strategy for LINC01260 was adopted, Oil red O staining and semiquantitative analysis were combined to explore whether the GOF of LINC01260 affects LO2 cell steatosis. CeRNA-based bioinformatics analysis of lncRNAs was performed, and the enriched mRNAs were further verified. RXRB siRNAs were applied and verify its role in LINC01260 regulated OA-induced hepatocytes steatosis.

**Results:**

Lipid droplets of different sizes were observed in the cells of the OA group. Absorbance in the OA group was significantly increased after isopropanol decolorization (*P* < 0.05). Compared with those in the control group, there were 648 lncRNAs with differential expression greater than 1 time in the OA group, of which 351 were upregulated and 297 were downregulated. Fluorescence quantitative PCR showed that the expression of LINC01260 in the OA group was downregulated by 0.35 ± 0.07-fold (*P* < 0.05). The formation of lipid droplets in LO2 cells of the LINC01260 GOF group decreased significantly (*P* < 0.05). CeRNA analysis indicated that the mRNA levels of RXRB, RNPEPL1, CD82, MADD and KLC2 were changed to different degrees. Overexpression of LINC01260 significantly induced RXRB transcription (*P* < 0.05) and translation, and RXRB silence attenuated the lipids decrease induced by LINC01260 overexpression.

**Conclusion:**

The OA-induced NAFLD cell model has a wide range of lncRNA differential expression profiles. LINC01260 participates in the regulation of the lipid droplet formation process of NAFLD, and its overexpression can significantly inhibit the steatosis process of LO2 cells. Mechanistically, LINC01260 may act as a ceRNA to regulate the expression of RXRB, thereby affecting the adipocytokine signaling pathway.

**Supplementary Information:**

The online version contains supplementary material available at 10.1186/s12986-021-00634-4.

## Introduction

Nonalcoholic fatty liver disease (NAFLD) is a syndrome mainly characterized by diffuse hepatocyte bullous fat. The cause of NAFLD is very complicated, but alcohol and other clear liver damage factors are excluded [1]. NAFLD has become the most common chronic liver disease and the primary reason for abnormal liver biochemical indicators in health examinations in China [2], bringing a considerable health and economic burden to patients, families, and society.

Long noncoding RNAs (lncRNAs) are noncoding RNAs with lengths greater than 200 base pairs. Because of the lack of an open reading frame, lncRNAs do not encode proteins. LncRNAs can regulate many biological processes, including the cell cycle, apoptosis, and differentiation [3]. In recent years, many studies have confirmed that lncRNAs play an important role in the occurrence and development of NAFLD [4–7]. Emerging evidence suggests that lncRNAs may regulate hepatic lipid metabolism in NAFLD.

The most well-known lncRNA is H19, which is highly expressed in NAFLD. Previous studies showed that H19, as a miRNA decoy, targets miR-130a to regulate PPARγ expression and plays an important role in NAFLD [8]. Wang investigated the role of H19 in hepatic lipid metabolism and its potential association with NAFLD [9]. They found that exogenous overexpression of H19 in hepatocytes induced lipid accumulation and upregulated the expression of numerous genes involved in lipid synthesis, storage and breakdown. Mechanistically, H19 induces hepatic steatosis by activating the MLXIPL and mTORC1 networks in hepatocytes. In addition to H19, several other lncRNAs are involved in NAFLD pathogenesis, particularly in liver steatosis. Another lncRNA is Gm15622, which stimulates SREBP-1c expression and hepatic lipid accumulation by sponging miR-742-3p in mice [10]. Additionally, the lncRNA Gm12664-001 was demonstrated to attenuate hepatic lipid accumulation by negatively regulating miR-295-5p and enhancing CAV1 expression in AML12 cells [11]. In addition, the lncRNA Gomafu upregulates the expression of the transcription factor FoxO1 by sponging miR-139-5p, thereby promoting insulin resistance in the liver, reducing the lipolysis ability of the liver, and increasing lipid deposition in NAFLD [12]. However, the mechanism of action of lncRNAs in NAFLD has not yet been fully elucidated. Identifying additional novel lncRNAs involved in NAFLD is still important.

Competing endogenous RNAs (CeRNA) hypothesis RNA transcripts can crosstalk by competing for common microRNAs, with microRNA response elements (MREs) as the foundation of this interaction [13]. These RNA transcripts have been termed as competing endogenous RNAs—ceRNAs [14]. Any RNA transcript with MREs might act as ceRNA, and ceRNAs include pseudogene transcripts, lncRNAs, circRNAs and mRNAs, these transcripts can compete for the same microRNA response elements (MERs) to regulate mutually. To find potential target of microRNAs, the target/microRNAs is usually predicted with home-made miRNA target prediction software based on TargetScan & miRanda [15, 16].

In this article, we established lncRNA profile in an in vitro model of NAFLD in cultured LO2 cells using oleic acid (OA) stimulation. Six lncRNAs were selected to explore their roles in NAFLD. Then, LINC01260 (ENST00000255183) was investigated in detail, and its potential mechanism as a ceRNA in NAFLD was investigated. Finally, LINC01260 was proposed as a key hepatic steatosis regulator, and its mechanism is probably related to the upregulation of RXRB expression as ceRNA. The present study provides theoretical support for the clinical value of LINC01260 in NAFLD.

## Materials and methods

### Reagents

The normal human liver cell line LO2 (HL-7702) was purchased from Wuhan Procell Life Science & Technology Co., Ltd. (China). An RNA extraction kit was obtained from Shanghai Yishan Biotechnology Co., Ltd. (China). A RevertAid First Strand cDNA Synthesis Kit (#k1622) was purchased from Thermo Fisher Scientific (USA). FastStart Essential DNA Green Master Mix was obtained from Roche (Switzerland). Lipofectamine 2000 transfection reagent was purchased from Invitrogen (USA); the lncRNA overexpression plasmid, negative control plasmid, and lncRNA primers were designed and synthesized by Biosune Biotechnology (Shanghai) Co., Ltd. (China).

### Cell culture and hepatic steatosis model induction

LO2 cells were cultured in RPMI-1640 medium supplemented with 10% FBS, 100 units/mL penicillin, and 100 µg/mL streptomycin. When the cell density reached approximately 60%, LO2 cells were exposed to different doses of OA (10, 20, 30, 40, 50, 60 μg/mL) or DMSO for 48 h. The medium were changed every 24 h.

### Oil red O staining

After steatosis induction, LO2 cells were fixed with 4% tissue/cell fixative for 10 min and then stained for 30 min with Oil red O (0.6 mg/mL) under room temperature and dark conditions. After staining, 60% isopropanol was added for 1 min to remove the background signal. An inverted microscope (Olympus, Japan) was used to observe droplets in cells. Then, decolorization was performed using 100% isopropanol for 10 min at 37 °C. The OD value of the decolorization solution was detected by a microplate reader at a wavelength of 510 nm.

### LncRNA sequencing

Eight samples were collected consisting of 4 control and 4 experimental samples, labeled control (ctrl) 1, ctrl 2, ctrl 3, ctrl 4, test 1, test 2, test 3, and test 4. Control group means LO2 cells exposed to DMSO for 48 h; test group means LO2 cells exposed to OA (50 μg/mL) for 48 h. Then, cells were harvested in Trizol reagent and all samples were sent to Shanghai Kangcheng Biotechnology Co., Ltd. for eukaryotic circular sequencing to detect the expression changes in lncRNAs in the NAFLD cell model. The general protocol, including cell treatment, RNA extraction, RNA library establishment, qPCR quantification and sequencing, was described in published work (*Lipids Health Dis*. 2021; 20(1):39).

### Quantitative real-time PCR (qPCR)

An RNA extraction kit was used to extract total RNA as protocol. Microarray data were validated by qPCR, with β-actin as a control. The primer sequences are detailed in Additional file 1: Table S1.

### Cell transfection

Overexpression plasmids of target lncRNAs were constructed with pcDNA3.1 as the vector. Lipofectamine 2000 was the transfection reagent. The detailed steps were performed according to the manufacturer’s protocols. Cells were harvested 24 h after transfection for the luciferase assay. The expression of target lncRNAs was detected using qPCR. All transfection experiments were performed in triplicate.

### CeRNA network prediction for the lncRNA LINC01260

Further bioinformatics analysis was conducted on the lncRNA LINC01260 to construct a ceRNA network and predict its downstream mRNAs. The intersection between the identified downstream mRNAs and an mRNAs profile based on LO2 sequencing results (*Lipids Health Dis*. 2021; 20(1):39) was performed using the https://bioinfogp.cnb.csic.es/tools/venny/ website. Generally, to find potential target of microRNAs, the target/microRNAs is predicted with home-made miRNA target prediction software based on TargetScan & miRanda. Besides a measure with the number of common microRNAs, a hypergeometric test is executed for each ceRNA pair separately. In the present study, we first get mRNAs intersection based on bioinformatics mRNA prediction data and mRNA profile from RNA Sequencing. Then, target miRNAs were predicted for the intersection mRNAs. Then, qRT-PCR verification was performed to verify the resulting mRNAs.

### RXRB siRNAs sequences

Three specific siRNAs for RXRB were designed, and they are RXRB siRNA-1187, siRNA-1311, siRNA-1512. Meaningless control sequences were synthesized as negative control. These siRNAs were transfected into LO-2 cells, respectively, to investigate the effect of RXRB silence to OA-induced steatosis. The siRNAs sequences were as follows:

siRNA-1187: Sense (5’–3’)-GACCCUGUGACUAACAUCUTT
  Antisense (5’–3’)-AGAUGUUAGUCACAGGGUCTT
siRNA-1311: Sense (5’–3’)-GCUGGAAUGAACUCCUCAUTT
  Antisense (5’–3’)-AUGAGGAGUUCAUUCCAGCTT
siRNA-1512: Sense (5’–3’)-GGGCAAUCAUUCUGUUUAATT
  Antisense (5’–3’)-UUAAACAGAAUGAUUGCCCTT

### Statistical analysis

All data in this study are presented as the mean ± SD, and SPSS 19.0 software was used for the statistical analysis. Differences between two groups were compared using Student's *t*-test. Analysis of variance (ANOVA) was used to compare the data of three or more groups. The *q* test was used for further pairwise comparisons. A value of *P* < 0.05 was considered statistically significant.

## Results

### A hepatic steatosis model was established in cultured LO2 cells

**Fig. 1:**
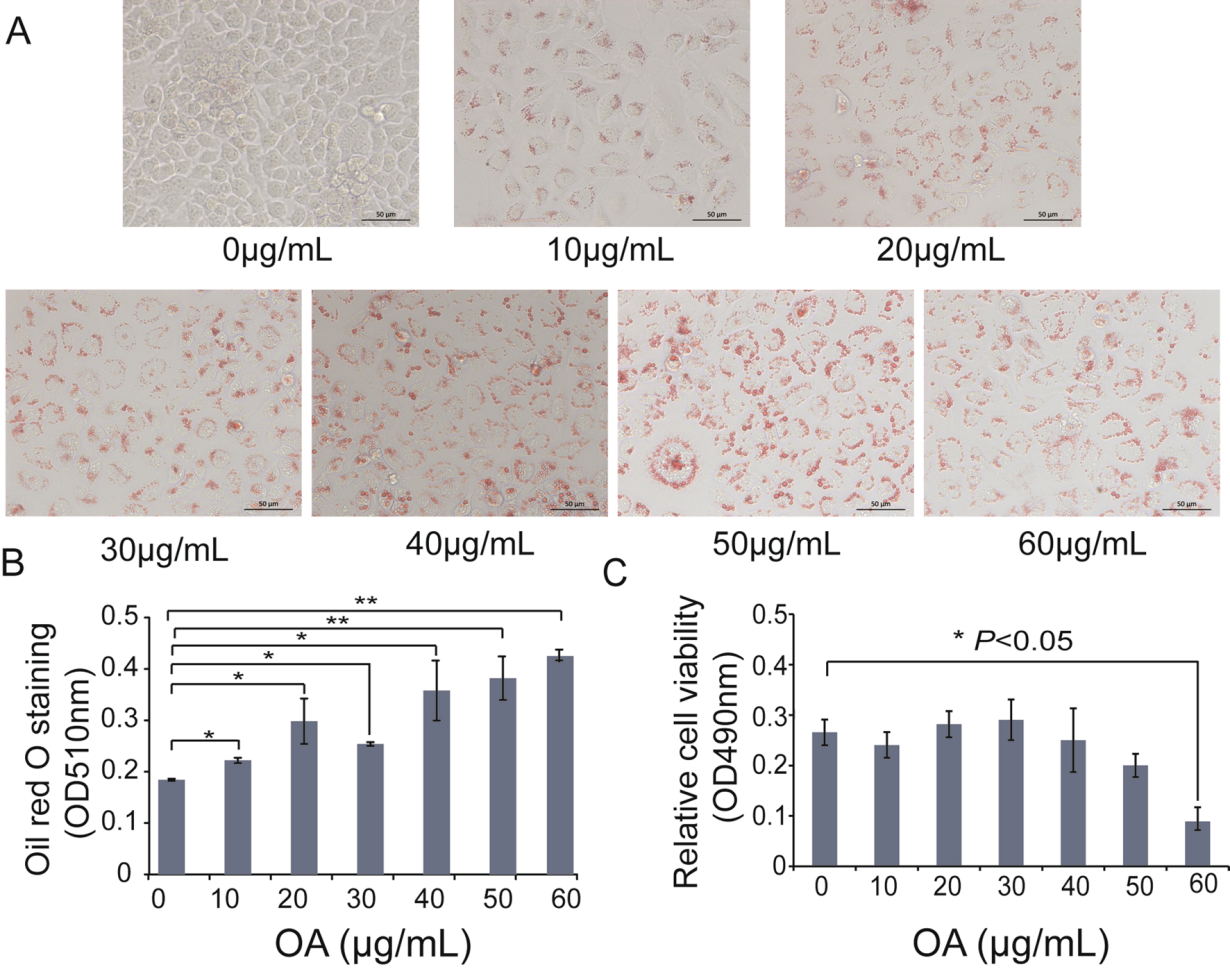
Oleic acid-induced cell model of hepatocyte steatosis. **A** Lipid accumulation was observed with oil red O staining under light microscopy (400 ×). **B** Semi-quantitative analysis of Oil red O staining (*P < 0.05, **P < 0.01). **C** Relative cell viabilities detection based on MTT method

A hepatic steatosis model was established using cultured LO2 cells by OA induction. Different doses of OA (10, 20, 30, 40, 50, 60 μg/mL) were tested, and Oil red O staining showed considerable deposition of lipid droplets in the cells of the OA group compared with the control group in an OA dose-dependent manner (Fig. 1a). Moreover, absorbance at 510 nm in the OA group increased significantly after isopropanol decolorization, indicating increased hepatic lipid accumulation (Fig. 1b). Cell viability of the OA group treated with a dose was less than 50 μg/mL showed no statistically significant decrease compared with the control group (Fig. 1c), indicating that 50 μg/mL OA treatment is the best dose for hepatic steatosis induction.

### LncRNA profile changes in a hepatic steatosis model of cultured LO2 cells

**Fig. 2:**
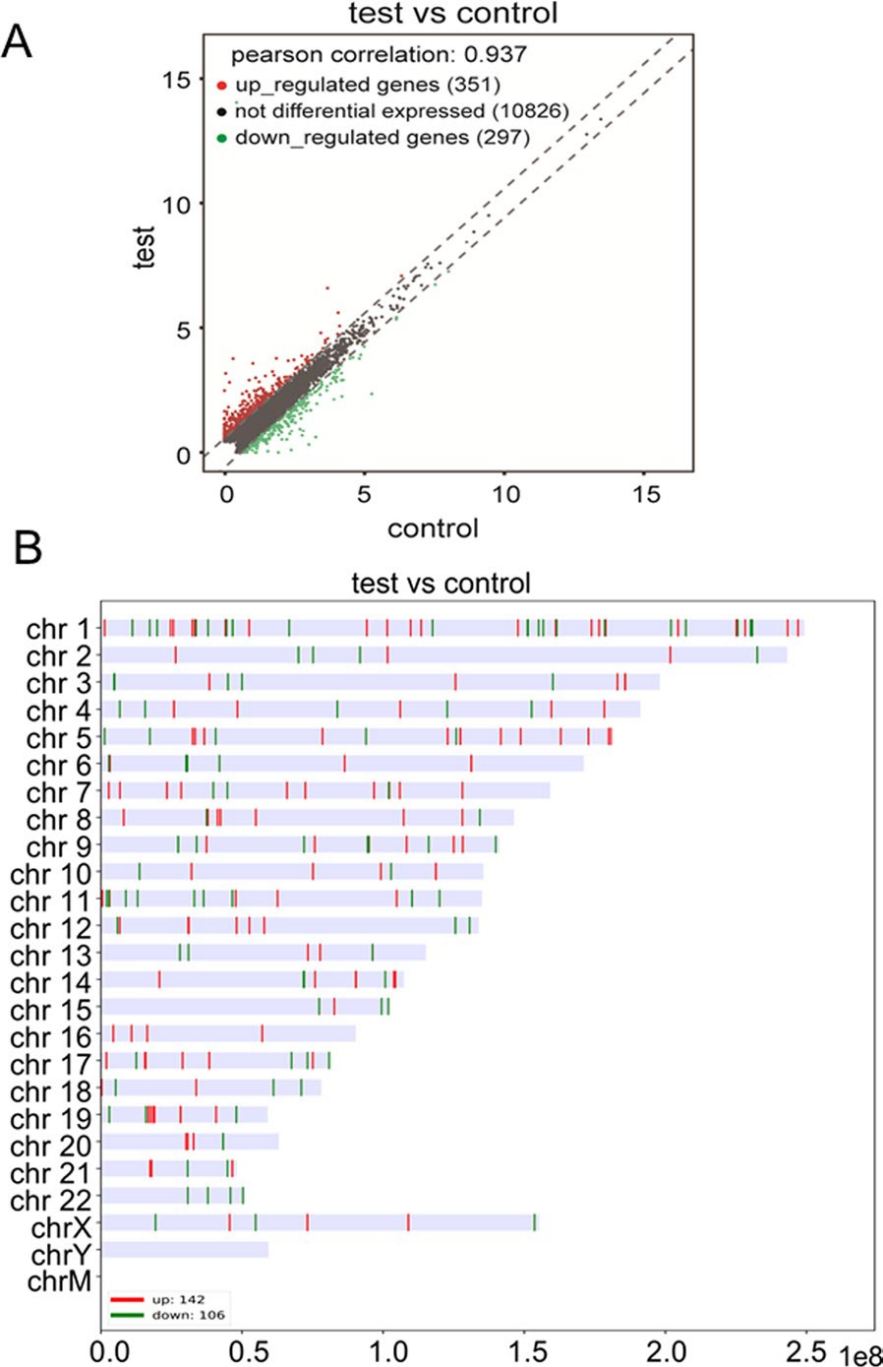
Profiles of lncRNAs and Karyogram depicting genomic localization of the genes in NAFLD cell model. **A** The scatter plot showed general profiles changes in lncRNAs between hepatocyte steatosis model and control cells. **B** Karyogram depicting genomic localization of the genes from co-expressed gene set

Using eukaryotic circular sequencing techniques, we detected the expression profiles of lncRNAs. The general profile information is shown in Fig. 2. The scatter plot shows that 297 lncRNAs were downregulated and 351 lncRNAs were upregulated in the LO2 steatosis model (Fig. 2a). Then, a karyogram depicting the genomic localization of the host genes of lncRNAs was generated. Further analysis revealed that the upregulated and downregulated lncRNAs were distributed on all chromosomes with the exception of chromosome Y. Chromosomes 1, 5 and 11 contained more lncRNAs than the other chromosomes (Fig. 2b).

### General informations of top 20 upregulated and top 20 downregulated lncRNAs

**Fig. 3:**
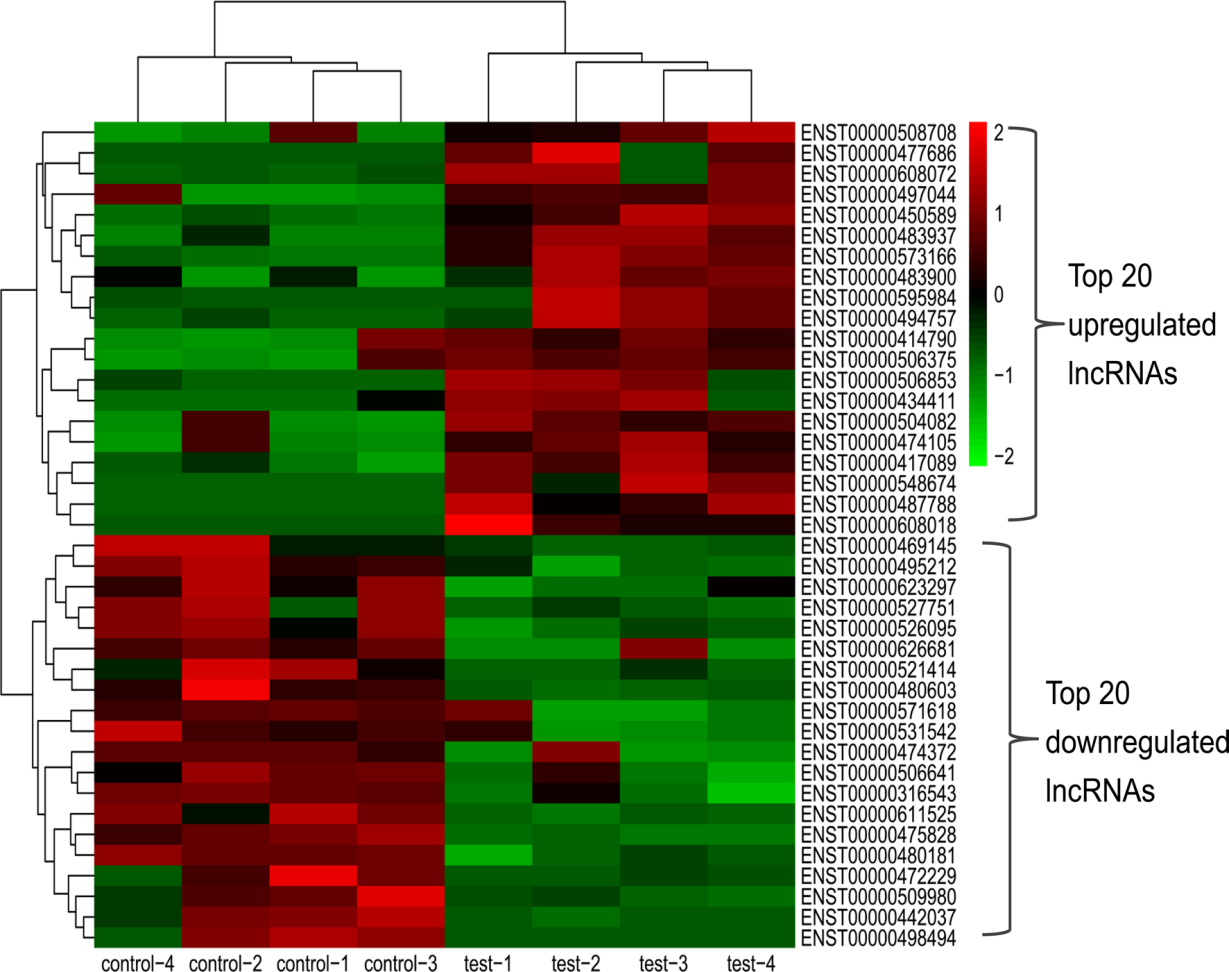
The heatmap of top 20 upregulated and top 20 downregulated lncRNAs. The top 20 upregulated and top 20 downregulated lncRNAs changed in oleic acid-induced hepatocyte steatosis model were scanned out and heatmap was produced

The top 20 upregulated and top 20 downregulated lncRNAs are scanned. Figure 3 showed the heatmap of the scanned top 20 upregulated and top 20 downregulated lncRNAs. All the fold changes of scanned lncRNAs were higher than twofold, with *P* valus less than 0.05. General informations of these lncRNAs were listed in Table 1, including lncRNA_id, associated_gene_name, log_2_FC, fold change, and *p* value.

**Table 1:** The details of top 20 up-reguated and down-regulated lncRNAs under oleic acid treatment by RNA sequencing

lncRNA_id	Associated_gene_name	log2FC	Fold_Change	*p*_value	lncRNA_id	Associated_gene_name	log2FC	Fold_Change	*p*_value
Up-reguated					Down-reguated				
ENST00000487788	MEAF6	3.1242	8.7195	0.0000	ENST00000442037		−2.9296	0.131	0.0064
ENST00000414790		2.8985	7.4566	0.0358	ENST00000472229	MRPS18B	−2.7050	0.1533	0.0426
ENST00000497044	SHTN1	2.5531	5.8689	0.0290	ENST00000521414	NDRG1	−2.6551	0.1587	0.0076
ENST00000477686	MRPL20	2.4789	5.5747	0.0261	ENST00000475828	MEAF6	−1.9648	0.2561	0.0000
ENST00000506375	SLAIN2	2.0151	4.0421	0.0211	ENST00000498494	SELENBP1	−1.9296	0.2625	0.0242
ENST00000450589	ZBTB37	1.9146	3.7701	0.0003	ENST00000571618	TBCD	−1.7755	0.2921	0.0391
ENST00000595984	COPE	1.8744	3.6666	0.0249	ENST00000509980	EXOSC9	−1.7089	0.3059	0.0094
ENST00000504082		1.7068	3.2644	0.0198	ENST00000626681	BACH1	−1.5875	0.3327	0.0430
ENST00000506853	AGA	1.6727	3.1882	0.0170	ENST00000469145	HDGF	−1.3972	0.3796	0.0148
ENST00000483937	MAPKAP1	1.6650	3.1712	0.0017	ENST00000527751	PRR5L	−1.1769	0.4422	0.0270
ENST00000483900	AHCTF1	1.6037	3.0394	0.0486	ENST00000526095	KCNQ1	−1.1363	0.4549	0.0021
ENST00000474105	IGF2BP3	1.5885	3.0074	0.0262	ENST00000611525		−1.1224	0.4593	0.0010
ENST00000417089		1.4379	2.7092	0.0031	ENST00000474372	UBAP2	−1.0733	0.4752	0.0484
ENST00000494757	WIPF2	1.4303	2.6950	0.0122	ENST00000531542	AMBRA1	−1.0705	0.4761	0.0168
ENST00000608018		1.3741	2.5920	0.0016	ENST00000480603	PPIA	−1.0677	0.4770	0.0011
ENST00000548674	ARHGAP9	1.3432	2.5372	0.0019	ENST00000506641	CLPTM1L	−1.0613	0.4791	0.0259
ENST00000434411	SEC14L1	1.3218	2.4998	0.0455	ENST00000480181	CLIC3	−1.0504	0.4828	0.0009
ENST00000608072	BACH1	1.3184	2.4939	0.0254	ENST00000495212	HDGF	−1.0479	0.4836	0.0042
ENST00000573166	HIC1	1.2892	2.4439	0.0000	ENST00000316543	AACS	−1.0453	0.4845	0.0105
ENST00000508708	CSNK1G3	1.2802	2.4287	0.0385	ENST00000623297		−1.0229	0.4921	0.0142

### Overexpression of LINC01260 inhibited hepatic steatosis

**Fig. 4:**
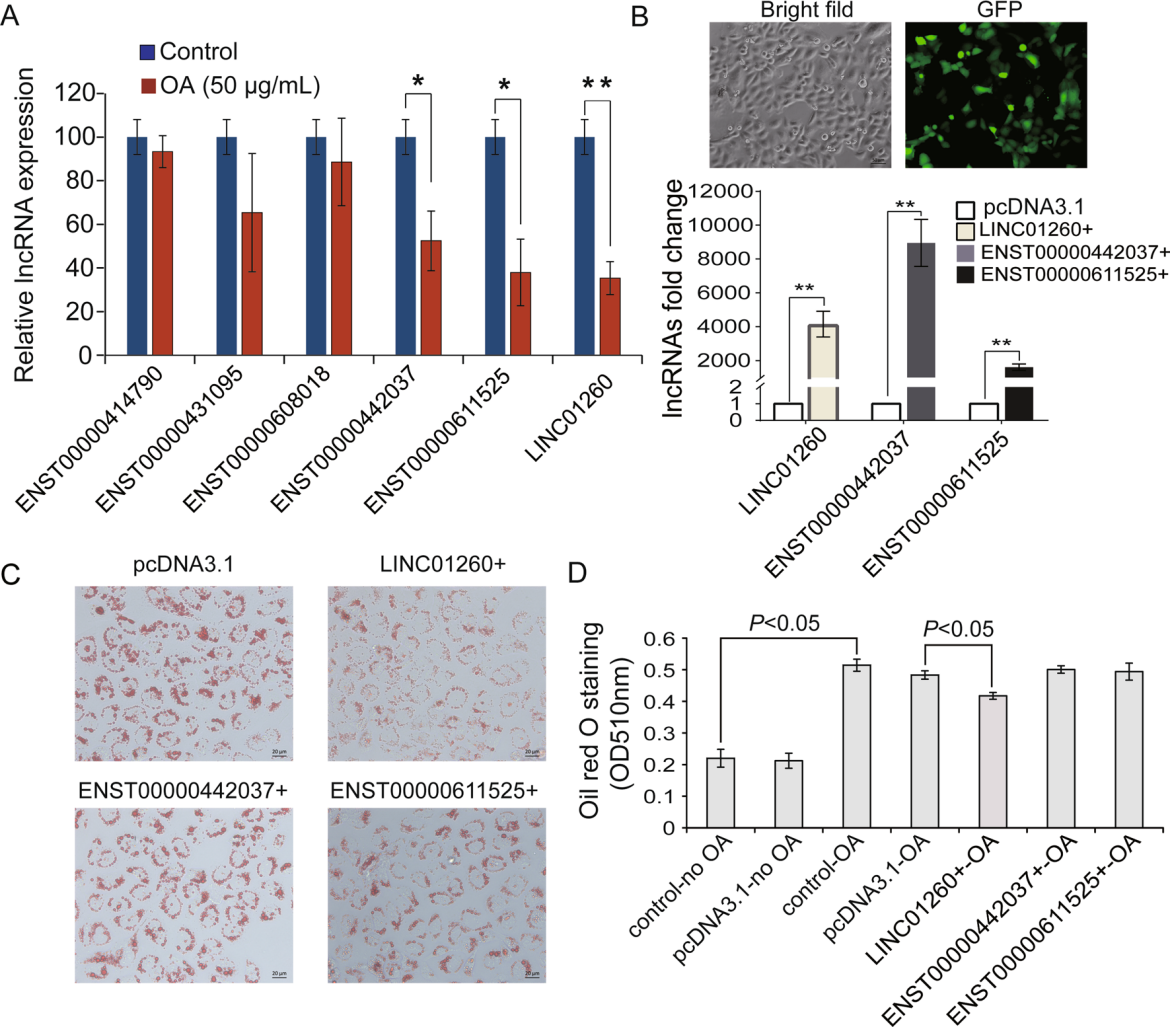
Overexpression of LINC01260 inhibits oleic acid-induced lipid accumulation in LO2 cells. **A** Six selected differentially expressed lncRNAs were validated by qPCR, with three of them consistent to sequencing. **B** Overexpressions of three lncRNAs were assayed with GFP fluorescence after transfection for 24 h. **C** Lipid accumulation after overexpression of three lncRNAs was detected by Oil red O staining. **D** Semi-quantitative analysis of Oil red O staining (*P < 0.05)

Based on the fold change and *p* value, six intergenic lncRNAs of interest, namely, lncRNA ENST00000414790, lncRNA ENST00000431095, lncRNA ENST00000608018, lncRNA ENST00000442037, lncRNA ENST00000611525 and LINC01260, were selected for qPCR verification. The results showed that LINC01260, ENST00000442037, and ENST00000611525 were downregulated by 0.35 ± 0.07, 0.52 ± 0.13, and 0.38 ± 0.15, respectively, compared to their expression in the control group (Fig. 4a), which was consistent with the sequencing results (*P < 0.05, **P < 0.01).

Next, we overexpressed the indicated three lncRNAs to investigate whether they could affect hepatocyte steatosis. Fluorescence microscopy showed that the transfection efficiency of the plasmids was approximately 80% (Fig. 4b). In comparison with those in the control, the levels of LINC01260, lncRNA ENST00000442037, and lncRNA ENST00000611525 in LO2 cells transfected with the overexpression plasmids were increased by (4151.36 ± 1316.90) %, (8946.66 ± 2405.24) %, and (1605.99 ± 329.68) %, respectively (Fig. 4b). Therefore, these expression plasmids were effective and were used for further experiments.

Then, Oil red O staining was used to examine lipid accumulation. As shown in Fig. 4c, lipid droplet accumulation did not show much difference in LO2 cells treated with ENST00000442037 + and ENST00000611525 + , while lipid droplet accumulation was reduced in LINC01260 + cells compared with pcDNA3.1 cells. Semiquantitative analysis after isopropanol decolorization showed a significant decrease between pcDNA3.1 vector and LINC01260 + groups under OA treatment (Fig. 4d) (*P* < 0.05). Compared with non-OA treated group (0.22 ± 0.028), OA treatment induced hepatocytes steatosis, with Oil red O absorbance value increased significantly (0.51 ± 0.02). Compared with non-treated vector negative control group (0.22 ± 0.023), OA treated vector control group showed obvious steatosis, with Oil red O absorbance value increased significantly (0.48 ± 0.013). The overexpression of LINC01260 leading to a decrease in Oil red staining (0.42 ± 0.01). These results indicated that overexpression of LINC01260 could inhibit hepatocyte steatosis.

### The LINC01260-RXRB axis may be responsible for hepatic steatosis

**Fig. 5:**
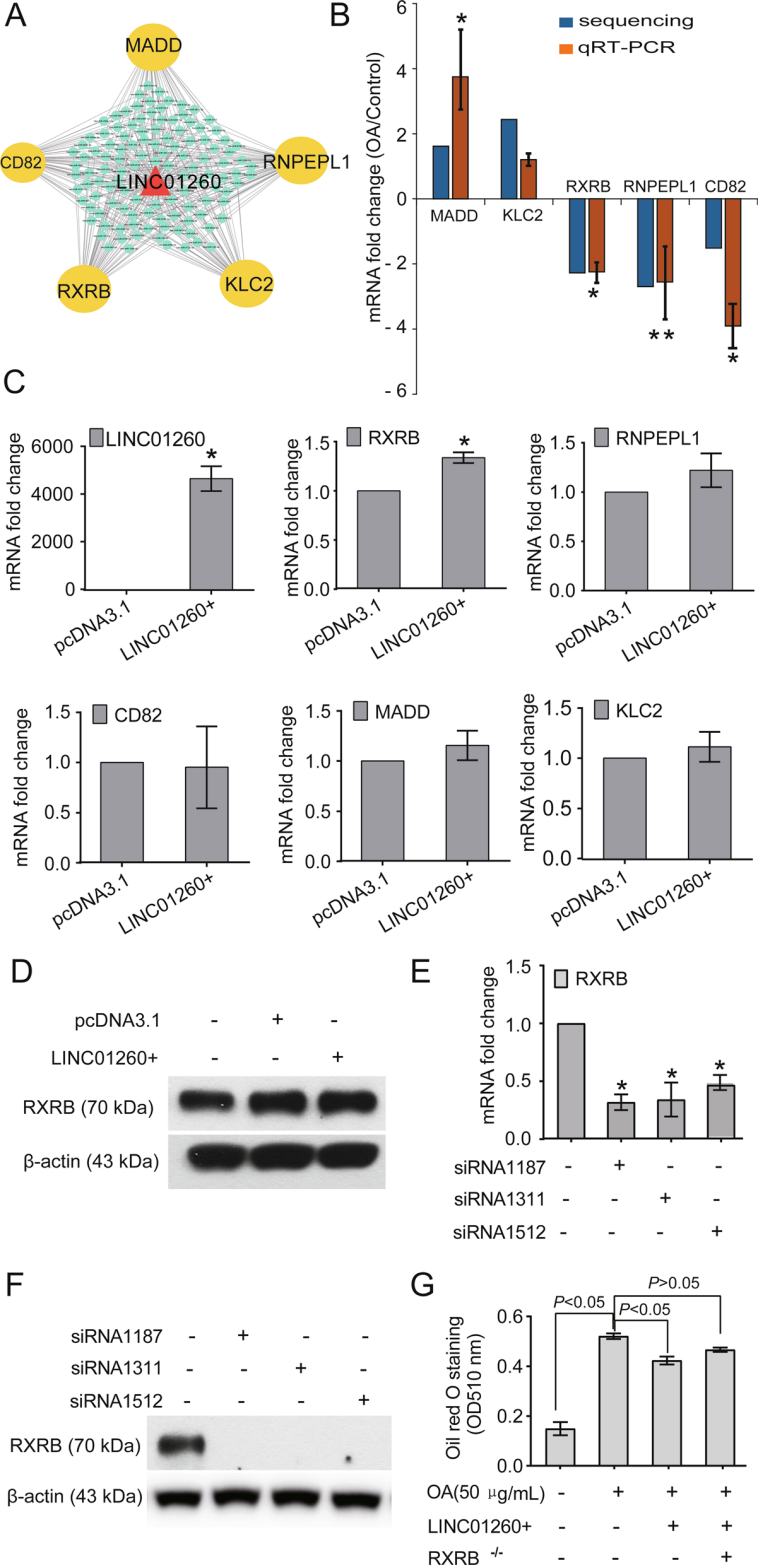
CeRNA network analysis of LINC01260. **A** Bioinformatic analysis of mRNAs co-expressed with LINC01260. RXRB, RNPEPL1, CD82, MADD and KLC2 were enriched. **B** Validation of co-expressed mRNAs by qPCR, and all were consistent with sequencing (*P < 0.05, **P < 0.01). **C** Overexpression of LINC01260 (LINC01260 +) increased the expression RXRB significantly (*P < 0.05) compared with pcDNA3.1 groups. **D** Western blots proved the upregulation trends of RXRB protein in LINC01260 + cells. **E** RXRB siRNAs decreased RXRB gene expression significantly (*P < 0.05). **F** RXRB siRNAs decreased RXRB protein expression significantly (*P < 0.05). **G** RXRB siRNA attenuated the decrease of lipids induced by LINC01260 overexpression

Further bioinformatics analysis of LINC01260 was performed, and mRNAs regulated by this lncRNA acting as a ceRNA were predicted. In the present study, there were 241 mRNAs forming a ceRNA network with LINC01260. We compared the sequencing mRNA expression profile with the 241 mRNAs in the ceRNA network and found that there were 5 mRNAs, namely, RXRB, RNPEPL1, CD82, MADD and KLC2, with significant differences. Further, the ceRNA network showed that LINC01260 could indirectly regulate the expression of these mRNAs by competing with 119 miRNAs (Fig. 5a).

To confirm the sequencing results of these mRNAs, qRT-PCR was conducted and showed that RXRB, RNPEPL1 and CD82 were downregulated by (2.24 ± 0.35), (2.55 ± 1.03), and (3.91 ± 0.92) fold, respectively, while MADD and KLC2 were upregulated by (3.75 ± 1.36) and (1.20 ± 0.19) fold, respectively, compared with the control group, which was consistent with the sequencing results (Fig. 5b).

Then, the influence of LINC01260 overexpression (LINC01260 +) on RXRB, RNPEPL1, CD82, MADD and KLC2 expression was assessed. The results showed that LINC01260 overexpression significantly increased the mRNA level of RXRB compared to that in the pcDNA3.1 group (*P < 0.05), with RNPEPL1, CD82, MADD and KLC2 showing no significant alteration (*P* > 0.05) (Fig. 5c). Western blots detection showed similar unregulated trends of RXRB protein in LINC01260 + cells (Fig. 5d).

Further, RXRB siRNAs effect was identified by qPCR and Western blots, both gene transcription (Fig. 5e) and protein expression (Fig. 5f) showed significant decrease in RXRB. In Oil red O staining, RXRB silence attenuated the decrease of lipids induced by LINC01260 overexpression, with no significant change between RXRB siRNAs group and control group (*P* > 0.05) (Fig. 5g).

## Discussion

With the improvement of human living standards and changes in dietary structure, the incidence of NAFLD has been increasing yearly. Studies on NAFLD have also received increasing attention. To date, there is no clinically effective drug for the treatment of NAFLD. The main principle of prevention and treatment is to choose liver protection and fat-removing drugs on the basis of diet adjustment and exercise [17, 18]. Scholars at home and abroad are actively exploring the complex pathogenesis of NAFLD. Considering that the increase in free fatty acids is one of the important mechanisms of NAFLD and that OA has the highest content of free fatty acids, OA was chosen to establish the NAFLD cell model.

Although lncRNAs do not encode proteins, they have important functions in gene expression regulation, such as transcription level regulation, chromosome silencing and modification. At present, studies have found that lncRNAs play an important role in NAFLD and are involved in lipid metabolism, β-oxidation, IR, inflammation, and liver fibrosis in the process of NAFLD [4–7]. Using lncRNA sequencing technology, researchers analyzed the liver tissues of NAFLD patients and a NAFLD mouse model induced by a high-fat diet [19, 20] and found differences in the expression profiles of lncRNAs. To reduce interference factors such as the environment, individual differences and diet in animal NAFLD models and patient specimens, our study applied NAFLD cell models and then detected the differential expression profiles of lncRNAs in the NAFLD cell models. We identified a total of 648 differentially expressed lncRNAs in the OA group compared to the control group, of which 351 were upregulated and 297 were downregulated. Combined with the results of previous studies, these findings suggest that a variety of lncRNAs are involved in the occurrence and development of NAFLD.

We found that the expression of LINC01260 in the OA group was significantly downregulated, which was consistent with the sequencing results. LINC01260, also known as lncRNA ENST00000255183, is an intergenic lncRNA located on human chromosome 20. This lncRNA is poorly conserved, and its homologous sequence does not exist in mice or rats. At present, there are few studies on LINC01260. Researchers in South Korea conducted a genome-wide copy number variation study on osteoporotic fracture patients and healthy controls and found that one of the significantly related regions is located upstream of LINC01260, which provides an indication of the genetic factors associated with the risk of osteoporotic fractures [21]. Studies have found that patients with melanoma can be divided into high-risk groups and low-risk groups with different survival times by a molecular signature of six lncRNAs, including LINC01260, HCP5, PIGBOS1, RP11-247L20.4, CTA-292E10.6, and CTB-113P19.5 [22]. In addition, it has been reported that LINC01260 expression is downregulated in spinal glioma, and overexpression of LINC01260 targets CARD11 through the NF-κB signaling pathway to inhibit the proliferation, migration and invasion of spinal glioma cells [23].

There is no report on the role of LINC01260 in NAFLD. To explore the function of LINC01260 and the potential therapeutic value of LINC01260 for treating NAFLD, we constructed a LINC01260 overexpression plasmid. Interestingly, overexpression of LINC01260 significantly inhibited the fatty change process induced by OA in LO2 cells, suggesting that LINC01260 is closely related to lipid accumulation in NAFLD cell models.

Moreover, further competing endogenouse RNA (ceRNA) bioinformatics analysis predicted 5 mRNAs as targets, including RXRB, which was proved positively correlated with LINC01260. We found that RXRB expression was downregulated in NAFLD. RXRB is a member of the nuclear receptor superfamily of retinoic acid X receptors (RXRs) and is expressed in almost all tissues. RXRs have been reported to mediate lipid metabolism by forming dimers with PPAR [24]. Activating RXRs/PPARα increases the β-oxidation of fatty acids by upregulating the expression of the fatty acid oxidation rate-limiting enzyme SCAD [25]. Mechanistically, based on in vitro GOF experiments, we determined that overexpression of LINC01260 can positively regulate RXRB expression. Thus, it is conceivable that LINC01260 may regulate hepatic lipid homeostasis through ceRNA mechanism possibly involving the expression of RXRB. In NAFLD, LINC01260 was down regulated, so the miRNA binding with LINC01260 decreased. At the same time, the competing miRNA binding with RXRB increased. Since miRNA negatively regulate target RNA, so RXRB gene expression decreased under LINC01260 decreasing state. Thus, in NAFLD, LINC01260 positively regulate RXRB as ceRNA and regulate NAFLD process.

It should be noted that the fold changes for RXRB gene and protein expression are both small. Less than one fold change in transcription sometimes showed meaningless and no significant biological effect, although the difference showed significance. From transcription to translation, there are many regulators; any change could lead to different translation level. Also, for example, we detected FASN protein under LINC01260 overexpression, and found a decrease of FASN in LINC01260 overexpression group (in another work, data not shown here). It indicated that LINC01260 overexpression may influent hepatocytes steatosis from other mechanism. RXRB may not be the critical regulator related with LINC01260. Further efforts are needed to delineate the specific mechanisms.

It is undeniable that some limitations could be found for the present work. For example: (1) Oil red O staining is very generic for neutral lipid droplets. Only oleic acid, not other fatty acids (palmitic, palmitoleic, steric, or linoleic acid) were provided. Single component is hard to simulate in vivo state. Also, acute treatment of oleic acid for 48 h is different than chronic high fat conditions in vivo. Generally chronic high fat conditions in vivo are always accompanied with kinds of lipids changes, including saturated and unsaturated fatty acids. For example, in vivo high fat body usually includes high Triglyceride (TAG), Diglyceride (DAG), Phospholipids (PL), Phosphatidylethanolamine (PE), etc. It is the result of complex multi factor interaction, and single component is hard to simulate in vivo state. Our in vivo study in ongoing in another study.

(2) The present study investigated only steatosis as a biological endpoint, without investigation about cellular morphology (such as ballooning), inflammation or lipids content identification. The mechanism of NAFLD/NASH induced liver injury is considered to be a "two hit" phenomenon. The “first hit” includes inflammation and steotosis, which exacerbates liver sensitive to various "second hit" and lead to fibrosis [26]. Many studies proved that lipids plays critical function by inducing inflammation, cell injury and fibrosis [27]. Excessive lipid accumulation in liver is accompanied by series of histological changes from simple steatosis to non-alcoholic steatohepatitis, and will progress to cirrhosis with time [28]. Excessive lipid accumulation induced by the imbalance of liver lipid metabolism is the main cause of NAFLD. Additionally, more than 5% hepatocytes with lipids are the histological standards in NAFLD diagnosis. Also, inflammation is one of important endpoints in our research team, and another ongoing research work is underway. Over all, we focused on hepatocytes steatosis in the present study.

Taken together, the present study provided the function of LINC01260 in the regulation of the lipid droplet formation process of NAFLD. As far as our knowledge, it is novel in reporting LINC01260 in lipids accumulation. Also, the present study indicated an informative read for the mechanism of LINC01260 in lipids. These in vitro findings indicated the clinical value of LINC01260 in vivo, although largely keeps unknown about LINC01260 in vivo function. Overexpression of LINC01260 is of clinical value in NAFLD prevention and treatment.

## Conclusions

In summary, we demonstrated that LINC01260 expression was significantly downregulated in the NAFLD cell model. Overexpression of LINC01260 inhibited the steatosis process induced by OA in liver cells. Collectively, our findings strongly suggest that LINC01260 may play an important role in regulating hepatic lipid metabolism through RXRB regulation and may serve as a potential therapeutic target for NAFLD.

## Supplementary Information

**Additional file 1**. **Table 1.** Primer sequences for qPCR.

## Data Availability

All data generated or analyzed during this study are included in this published article or are available from the corresponding author on reasonable request.

## Abbreviations

NAFLD: Nonalcoholic fatty liver disease
lncRNAs: Long noncoding RNAs
OA: Oleic acid
GOF: Gain-of-function
ceRNA: Competing endogenous RNA
qPCR: Quantitative real-time PCR
