# Supplementary material for: The role and possible mechanism of the long noncoding RNA LINC01260 in nonalcoholic fatty liver disease
Source: Nutr Metab (Lond). 2022 Jan 12;19:3. doi: 10.1186/s12986-021-00634-4 (PMC8753873; doi:10.1186/s12986-021-00634-4)
Supplement: Supplementary file 1 — Additional file 1. Table 1. Primer sequences for qPCR. [file 12986_2021_634_MOESM1_ESM.docx]

Table 1. Primer sequences for qPCR

| Gene name | Sequences (5’-3’) | |
| --- | --- | --- |
| LINC 2160 | Forward | TCTCACCTGGAAGATGGGTC |
|  | Reverse | ATCTTCCTCTCATCCCGCAC |
| ENST00000414790 | Forward | CAAAGCCTCCACGACTCTGT |
|  | Reverse | ACTCACGCACACTCGTACTG |
| ENST00000431095 | Forward | ATGGTGCTACCCAGCTCAAG |
|  | Reverse | CACCTTCCAGAGCCGATTCC |
| ENST00000608018 | Forward | GGAGCACCTAAGAACTGGCA |
|  | Reverse | AGGTCTGCCATCTTGACACG |
| ENST00000442037 | Forward | CCCACAACATGAAAGCTTGGAAA |
|  | Reverse | TCCAGAGCCGATTCCTGAGT |
| ENST00000611525 | Forward | ACACGCCTCTGTCGTTGTAA |
|  | Reverse | CGGGAAAATGCCGCTTGAAA |
| RXRB | Forward | GCCCAAATGACCCTGTGACT |
|  | Reverse | AGTTCATTCCAGCCTGCCC |
| RNPEPL1 | Forward | GACAGCACTCTTCCTGGACC |
|  | Reverse | ATGCGGATCTCAGCGTTCAT |
| CD82 | Forward | GGAGAACCTGGGCATCATCC |
|  | Reverse | TGGGGACCTTGCTGTAGTCT |
| MADD | Forward | TGGAGAACATGACCGGAAGC |
|  | Reverse | CCTCACCTTCTTGCGGATGT |
| KLC2 | Forward | GTATGGCAAGAGGGGCAAGT |
|  | Reverse | CAGTGCCCGCCGATAGTAAT |
| β-actin | Forward | TGGATCAGCAAGCAGGAGTA |
|  | Reverse | TCGGCCACATTGTGAACTTT |
